# Side Effects of Radiographic Contrast Media

**DOI:** 10.1155/2014/872574

**Published:** 2014-06-02

**Authors:** Michele Andreucci

**Affiliations:** Department of Health Sciences, Magna Graecia University, Campus Salvatore Venuta, Viale Europa, Località Germaneto, 88100 Catanzaro, Italy

Radiographic contrast agents have been in use for over 60 years to improve the visibility of internal organs and structures in X-ray based imaging techniques such as radiography, angiography, and contrast-enhanced computed tomography scans and to perform cardiac catheterizations and percutaneous coronary interventions. Their use for imaging and intravascular intervention keeps increasing particularly in less healthy and older patients [1]. The risk of contrast-induced nephropathy (CIN) (or contrast-induced acute kidney injury (CI-AKI)) has been widely accepted in early medical literature and practice on the basis of a large number of uncontrolled observational studies [2]. The extent of toxicity of these agents has been questioned with the suggestion that the early literature had greatly overestimated the incidence of CIN and that CIN seems not to be common in patients with normal preexisting renal function, occurring more frequently in patients with renal impairment [3]. Nonetheless,* in vitro* cell culture studies have shown that all classes of contrast media are toxic causing a decrease in cell viability [4–9].

Thus, clinicians and radiologists, in clinical practice, ask themselves: “Are iodinated radiocontrast agents nephrotoxic? If so, what are the risk factors for CIN? And what are the appropriate procedures to prevent CIN?”

To answer these questions a special issue on side effects of radiographic contrast media was believed necessary, appointing expert authors to review the various aspects of contrast media nephrotoxicity. To help me in finding these expert authors, two outstanding Guest Editors were appointed: Richard Solomon (University of Vermont College of Medicine, Fletcher Allen Health Care, Burlington, VT, USA) and Adis Tasanarong (Nephrology Unit, Department of Medicine, Faculty of Medicine, Thammasat University, Rangsit Campus, Khlong Luang, Pathum Thani, Thailand).

For this volume we have invited authors who have been dealing with various aspects of contrast media toxicity. A wide array of topics are discussed in this special issue, including molecular mechanisms of renal cellular nephrotoxicity due to radiocontrast media; the considerable difference among iodinated contrast agents with regard to their osmolality and viscosity and the potential role of their osmolality and viscosity in the pathophysiology of CIN; the changes of renal hemodynamics as well as the renal tubular changes induced by iodinated contrast media; the crucial role of reactive oxygen species in causing CIN; and the role of intracellular Ca^2+^ and Na^+^/Ca^2+^ exchanger in the pathogenesis of CIN. Neither diabetes [10] nor multiple myeloma [11]* per se* can be considered main risk factors, but the important role of associated renal insufficiency and other clinical conditions in predisposing to CIN is discussed in depth. The current evidence on ACE-I/ARB therapy for patients undergoing procedures involving use of contrast media is also reviewed. The quest to find new strategies to prevent CIN has led to a recent clinical study suggesting the use of tocopherol [12]. In this special issue, there are also articles discussing the protective role against CIN of either isotonic sodium chloride solution or isotonic sodium bicarbonate solution, and nonpharmacological as well as pharmacological strategies for prevention of CIN. Finally, in one article the potential role of MBL (mannose-binding lectin, a pattern recognition protein of the lectin pathway of complement) in the pathogenesis of human CIN and the beneficial effects we may obtain in clinical practice by its inhibition with the C1 inhibitor, a potent MBL and lectin pathway inhibitor, are reviewed.
